# Supplementary material for: Prognostic Relevance of Nuclear Receptors in Relation to Peritumoral Inflammation and Tumor Infiltration by Lymphocytes in Breast Cancer
Source: Cancers (Basel). 2022 Sep 20;14(19):4561. doi: 10.3390/cancers14194561 (PMC9559250; doi:10.3390/cancers14194561)
Supplement: Supplementary file 1 [file cancers-14-04561-s001.zip › cancers-1828616-supplementary.pdf]

Supp. Figure S1: Kaplan-Meier survival analyses for cytoplasmic RXRα in relation to the level of peritumoral inflammation on overall survival (OS).

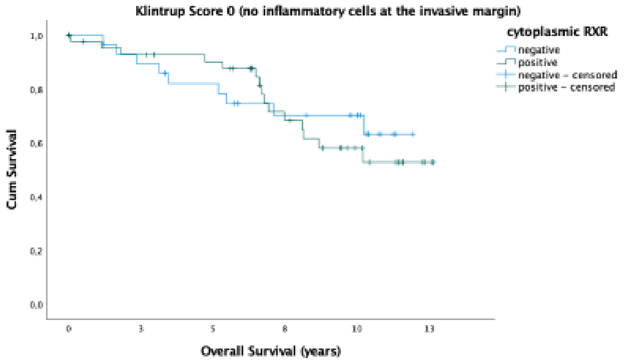

p = 0.681

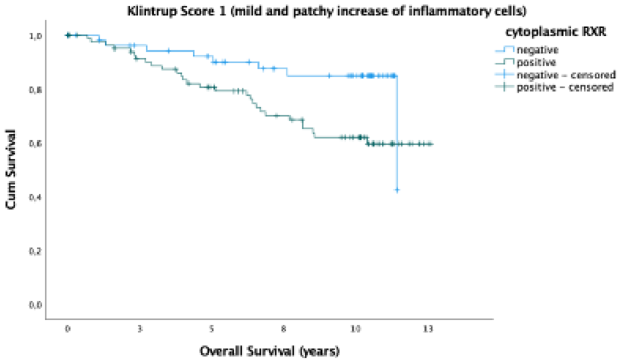

p = 0.021

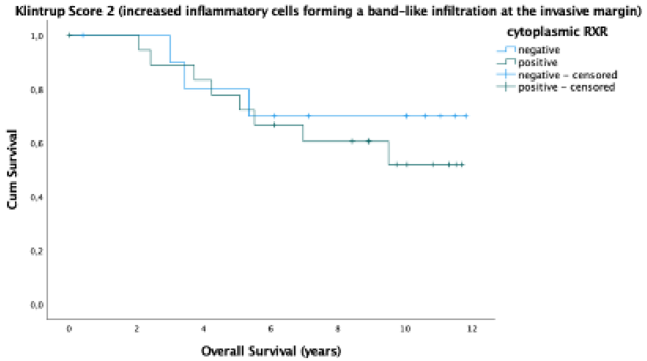

p = 0.401

Supp. Figure S2: Kaplan-Meier survival analyses for cytoplasmic LXR in relation to the level of peritumoral inflammation on overall survival (OS).

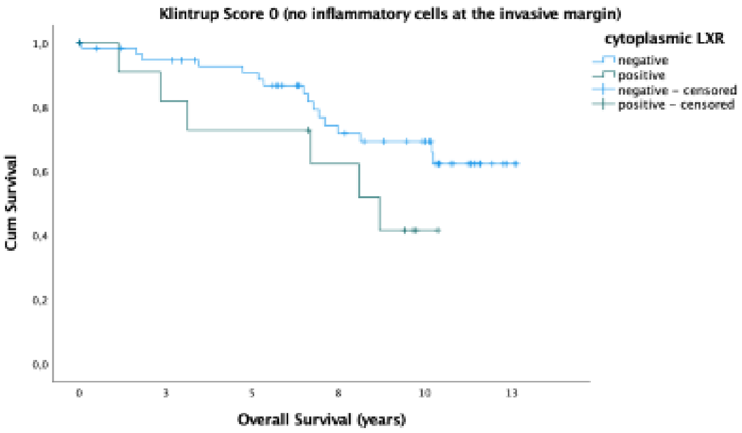

$p = 0.112$

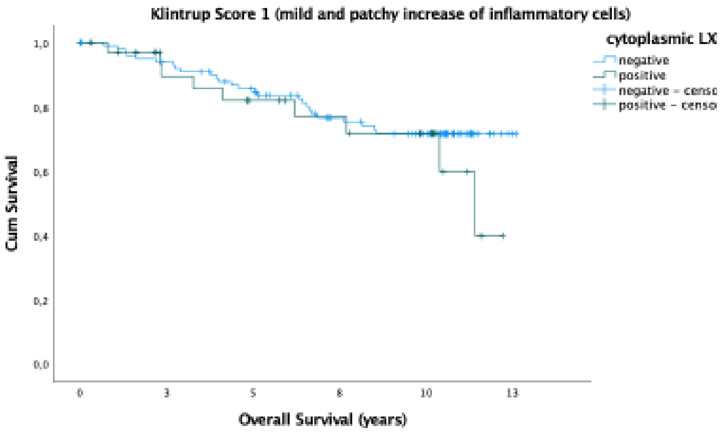

$p = 0.434$

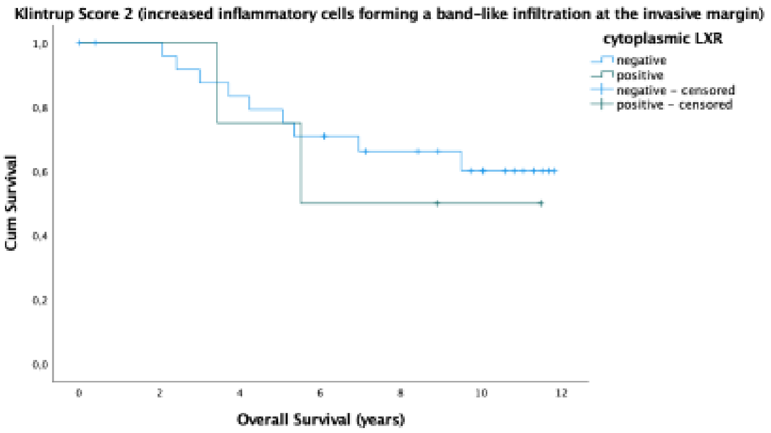

$p = 0.653$

Supp. Figure S3: Kaplan-Meier survival analyses for cytoplasmic AHR in relation to the level of peritumoral inflammation on overall survival (OS).

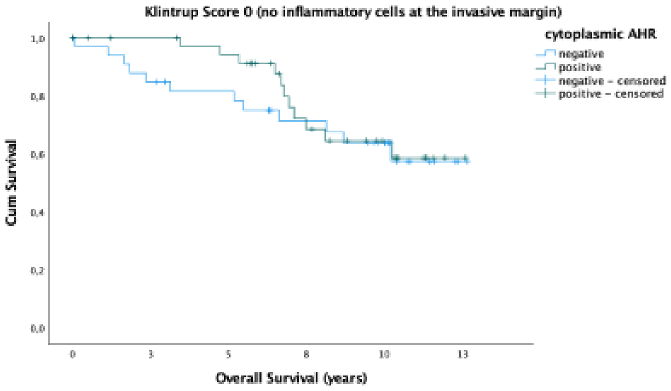

p = 0.595

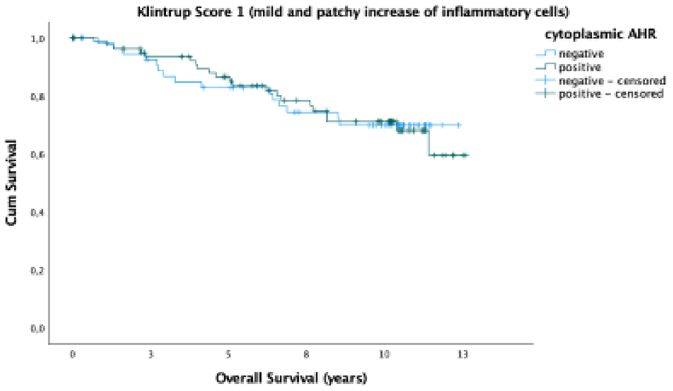

p = 0.961

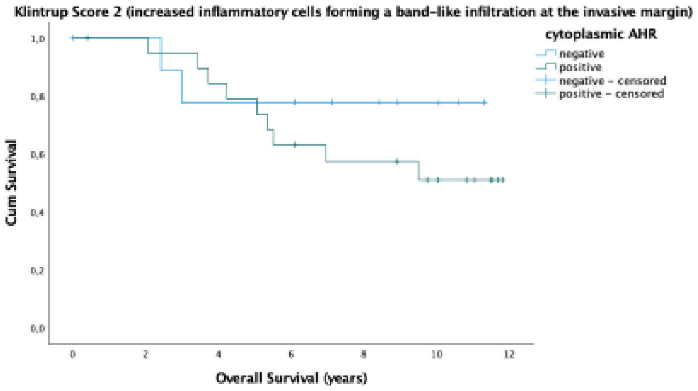

p = 0.347

Supp. Table S1

Clinical and pathological characteristics of all patients

**Clinical and Pathological Characteristics**	**n = 264**
Age, median (years)	57.54
Molecular subtype (IHC)	
Luminal A (Ki67 ≤ 14%)	140
Luminal B (Ki67 > 14%)	54
HER2 positive	19
HER2 non luminal	7
Triple negative	34
Unknown	10
Grade	
I	11
II	92
III	42
Unknown	119
Tumor size	
pT1	164
pT2	78
pT3	3
pT4	10
Unknown	9
Lymph node metastasis	
Yes	112
No	138
Unknown	14
ER status	
Positive	204
Negative	51
Unknown	9
PR status	
Positive	145
Negative	110
Unknown	9

Supp. Table S2: Correlation between Salgado and Klintrup Score

		Salgado			
		<10%	>10 and <30%	>30%	Total
Klintrup	0	60	14	0	74
	1	22	98	23	143
	2	0	0	30	30
Total		82	112	53	247

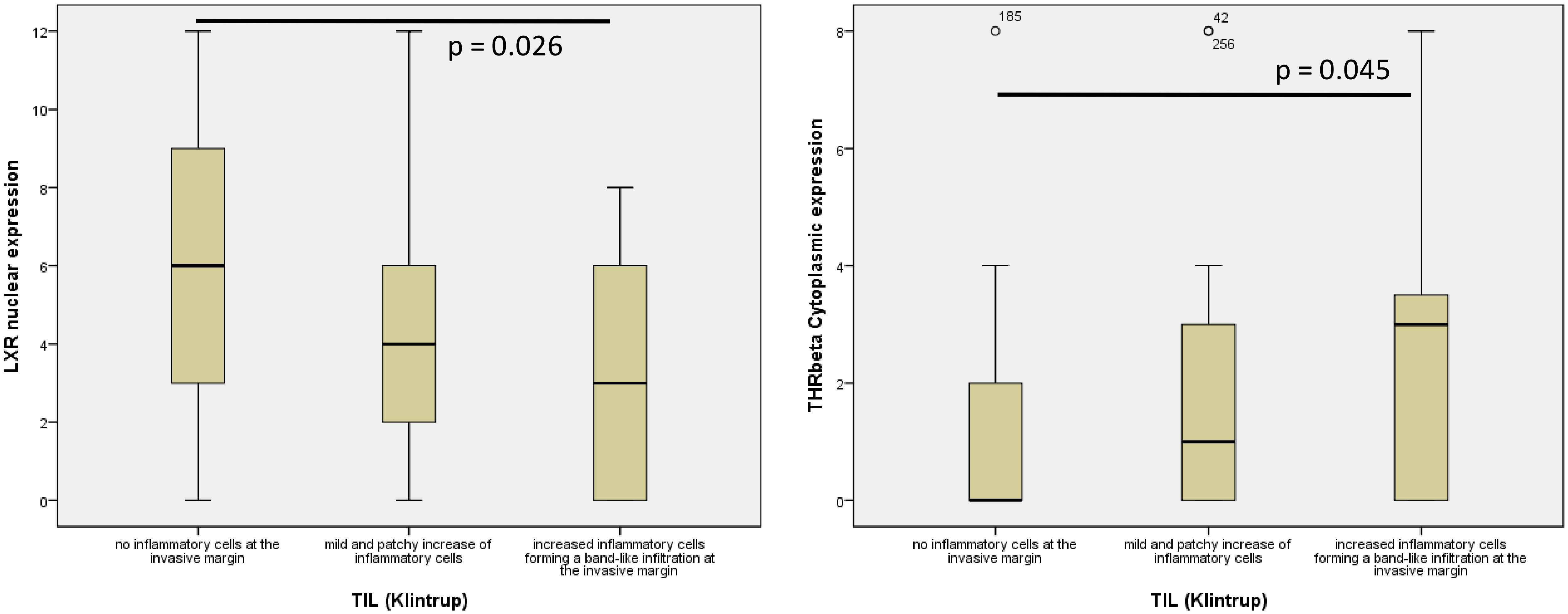

Supp. Figure S4: Expression of LXR in the nucleus is significantly decreased from cases with no peritumoral infiltration of immune cells to cases with increased numbers of infiltrating immune cells. In addition, expression of cytoplasmic expressed thyroid hormone receptor beta inreases from cases with no immune cell infiltration to cases with incresed infiltration of inflammatory cells.
